# Antioxidant Activity and Volatile and Phenolic Profiles of Essential Oil and Different Extracts of Wild Mint (*Mentha longifolia*) from the Pakistani Flora

**DOI:** 10.1155/2013/536490

**Published:** 2013-10-02

**Authors:** Tahseen Iqbal, Abdullah Ijaz Hussain, Shahzad Ali Shahid Chatha, Syed Ali Raza Naqvi, Tanveer Hussain Bokhari

**Affiliations:** Institute of Chemistry, Government College University Faisalabad, Faisalabad 38000, Pakistan

## Abstract

The antioxidant activity and free radical scavenging capacity of the essential oil and three different extracts of wildly grown *Mentha longifolia* (*M. longifolia*) were studied. The essential oil from *M. longifolia* aerial parts was isolated by hydrodistillation technique using Clevenger-type apparatus. The extracts were prepared with three solvents of different polarity (*n*-hexane, dichloromethane, and methanol) using Soxhlet extractor. Maximum extract yield was obtained with methanol (12.6 g/100 g) while the minimum with dichloromethane (3.50 g/100 g). The essential oil content was found to be 1.07 g/100 g. A total of 19 constituents were identified in the *M. longifolia* oil using GC/MS. The main components detected were piperitenone oxide, piperitenone, germacrene D, borneol, and **β**-caryophyllene. The total phenolics (TP) and total flavonoids (TF) contents of the methanol extract of *M. longifolia* were found to be significantly higher than dichloromethane and hexane extracts. The dichloromethane and methanol extracts exhibited excellent antioxidant activity as assessed by 2,2′-diphenyl-1-picrylhydrazyl (DPPH) free radical scavenging ability, bleaching **β**-carotene, and inhibition of linoleic acid peroxidation assays. The essential oil and hexane extract showed comparatively weaker antioxidant and free radical scavenging activities. The results of the study have validated the medicinal and antioxidant potential of *M. longifolia* essential oil and extracts.

## 1. Introduction

Free radicals are considered to initiate oxidation that leads to aging and causes diseases in human beings [1, 2]. Moreover, activated oxygen incorporates reactive oxygen species (ROS) which consists of free radicals (^1^O_2_, O_2_^∙−^, ^∙^OH, ONOO^−^) and nonfree radicals (H_2_O_2_, NO, and R–OOH) [3]. ROS are liberated by virtue of stress, and thus, an imbalance is developed in the body that damages cells in it and causes health problems [2, 4]. Moreover, oxidation in processed foods, enriched with fats and oils, during storage leads to spoilage and quality deterioration [5].

The use of synthetic antioxidants such as butylated hydroxyanisole (BHA), and butylated hydroxytoluene (BHT) and tertiary butylhydroquinone (TBHQ) have been restricted because of their carcinogenicity and other toxic properties [3, 6]. Thus, the interest in natural antioxidants has increased considerably. Natural antioxidants can be phenolic compounds (tocopherols, flavonoids, and phenolic acids) and carotenoids (lutein, lycopene, and carotene). Growing evidence has shown an inverse correlation between the intake of dietary antioxidants and the risk of chronic diseases such as coronary heart disease, cancer, and several other aging-related health concerns [1, 7].

Natural antioxidant compounds exhibit their antioxidant activity by various mechanisms including chain breaking by donation of hydrogen atoms or electrons that convert free radicals into more stable species and decomposing lipid peroxides into stable final products [1]. Different *in vitro *assays simply provide an idea of the protective efficacy of the test model. Thus it is necessary to use at least two methods depending on the expected antioxidant potential and/or on the origin of the substance. Most commonly used methods for the determination of antioxidant activity of plant essential oils and extracts are 2,2-di(4-*tert*-octaphenyl)-1-picrylhydrazyl (DPPH^∙^) radical scavenging assay, inhibition of linoleic acid peroxidation, and bleaching of **β**-carotene in linoleic acid system assays. DPPH radical scavenging assay is the most popular and frequently used for the determination of antioxidant activity of essential oils and plant extracts [1, 7, 8]. Bleachability of *β*-carotene in linoleic acid system is another simple, reproducible, and time efficient method for rapid evaluation of antioxidant properties [1, 7, 8]. Measurement of inhibition of linoleic acid peroxidation is also an effective method for the assessment of antioxidant activity of the plant samples.

*Mentha longifolia* (wild mint) belongs to genus *Mentha* (family Lamiaceae) and grows widely throughout the temperate regions of the world [8]. The different herbal and food products from *Mentha *species have been in use since ancient times for the treatment of heart burns, indigestion, colic, flatulence, coughs and flu, nausea, irritable bowel syndrome, gall-bladder and bile ducts, herpes, and certain skin infections including acne and pigmentation [7–9]. Research work on plants from different regions resulted in the innovation of biologically active substances [8, 10]. Therefore the study was conducted to investigate the chemical composition and antioxidant and antimicrobial activities of essential oil and three different extracts from *M. longifolia *native to dry region of Pakistan.

## 2. Materials and Methods

### 2.1. Collection and Pretreatment of Plant Material

Aerial parts of wild mint (*M. longifolia *L.) were collected during May-June from South Punjab, Pakistan. The specimens were further identified and authenticated by a taxonomist, Dr. Qasim Ali (Assistant Professor), Department of Botany, Government College University Faisalabad. Collected specimens were dried at 35°C in a hot air oven (IM-30 Irmec, Germany) and grinded to 80 mesh and stored in polyethylene bags at −4°C.

### 2.2. Chemicals and Reagents

Linoleic acid, 2,2′-diphenyl-1-picrylhydrazyl, gallic acid, Folin-Ciocalteu reagent, ascorbic acid, trichloroacetic acid, sodium nitrite, aluminum chloride, ammonium thiocyanate, ferrous chloride, ferric chloride, potassium ferricyanide, butylated hydroxytoluene (99.0%), and homologous series of C_9_–C_24_ *n-*alkanes and various reference chemicals used to identify the constituents were obtained from Sigma Chemical Co. (St. Louis, MO, USA). All other chemicals (analytical grade), that is, anhydrous sodium carbonate ferrous chloride, ammonium thiocyanate, chloroform, and methanol, used in this study were purchased from Merck (Darmstadt, Germany), unless stated otherwise. All culture media and standard antibiotic discs were purchased from Oxoid Ltd. (Hampshire, UK).

### 2.3. Isolation of Essential Oil

The oven-dried and ground fennel seeds (80 mesh) were subjected to hydrodistillation for 4 h, using a Clevenger-type apparatus. The obtained essential oil was dried over anhydrous sodium sulfate, filtered, and stored at −4°C until analyzed.

### 2.4. Preparation of Extracts

Ground (80 mesh) *M. longifolia *sample (100 g) was subjected to extraction for 4 h using Soxhlet unit. The plant materials were extracted in sequence with three solvents of different polarity, that is, *n*-hexane, dichloromethane and methanol. The extracts were concentrated under vacuum at 45°C, using a vacuum rotary evaporator (N–N Series, Eyela, Rikakikai Co. Ltd., Tokyo, Japan), and stored at −4°C until used for further analyses.

### 2.5. Analysis of the Essential Oil

#### 2.5.1. Gas Chromatography/Mass Spectrometry Analysis

The *M. longifolia* essential oil composition was determined on Agilent-Technologies (Little Falls, CA, USA) 6890N Network gas chromatographic (GC) system, equipped with an Agilent-Technologies 5975 inert XL Mass selective detector and Agilent-Technologies 7683B series autoinjector. Compounds were separated on HP-5 MS capillary column (30 m × 0.25 mm, film thickness 0.25 *μ*m; Little Falls, CA, USA). A sample of 1.0 *μ*L was injected in the split mode with split ratio 1 : 100. Helium was used as a carrier gas at a flow rate of 1.5 mL/min. For GC/MS detection, an electron ionization system, with ionization energy of 70 eV, was used. The column oven temperature was programmed from 80°C to 220°C at the rate of 4°C/min; initial and final temperatures were held for 3 and 10 min, respectively. Mass scanning range was 50–550 *m/z* while the injector and MS transfer line temperatures were set at 220 and 290°C, respectively. All quantifications were done by a built-in data-handling program of the equipment used (Perkin-Elmer, Norwalk, CT, USA). The composition was reported as a relative percentage of the total peak area.

#### 2.5.2. Compounds Identification

The components of the *M. longifolia *essential oil were identified by comparison of their retention indices relative to (C_9_–C_24_) *n-*alkanes either with those of published data or with authentic compounds [11, 12]. Compounds were further identified and authenticated using their complete mass fragmentation data compared to the NIST02.L and WILEY7n.L mass spectral libraries and published mass spectra and, wherever possible, by coinjection with authentic standards (*α*-pinene, *β*-pinene, limonene, cis-*β*-ocimene, *δ*-terpinene, 1,8-cineol, linalool, borneol, *α*-terpineol, thymol, piperitenone, piperitenone oxide, *β*-caryophyllene, germacrene D, calamenene, cis-jasmone, and caryophyllene oxide) [1, 13, 14].

### 2.6. Antioxidant Activity

#### 2.6.1. Determination of Total Phenolics (TP) and Total Flavonoids (TF) Contents

Amounts of total phenolics (TP) and total flavonoids (TF) in the *M. longifolia* extracts were determined using Folin-Ciocalteu reagent method and aluminum chloride colorimetric assay, respectively, as reported previously [15]. 

#### 2.6.2. DPPH Radical Scavenging Assay

2,2′-Diphenyl-1-picrylhydrazyl (DPPH) free radical assay was carried out to measure the free radical scavenging activity as reported previously [9]. Briefly, *M. longifolia* essential oil, extracts, piperitenone compound, and BHT concentrations in methanol (1–100 *μ*g/mL) were mixed with 2 mL of 90 *μ*M methanol solution of DPPH. After 30 min incubation period at room temperature, the absorbance was read at 517 nm. The scavenging (%) was calculated by the following formula:

(1) $$\begin{array}{c} \text{Scavenging}\left(\%\right)=100\times \left\{\frac{\left(A_{\text{blank}}-A_{\text{sample}}\right)}{A_{\text{blank}}}\right\}, \end{array}$$

where *A*_blank_ is the absorbance of the DPPH solution and *A*_sample_ is the absorbance of the extract solution. Extract concentration providing 50% scavenging (IC_50_) was calculated from the graph plotted between scavenging percentage and extract concentration.

#### 2.6.3. Antioxidant Activity Determination in Linoleic Acid System

The antioxidant activity of *M. longifolia *essential oil and extracts weas determined in terms of measurement of % inhibition of peroxidation in linoleic acid system following the reported method with some modification [16]. Essential oil and extracts (5 mg) were added to a solution mixture of linoleic acid (0.13 mL), 99.8% ethanol (10 mL), and 10 mL of 0.2 M sodium phosphate buffer (pH 7). Total mixture was diluted to 25 mL with distilled water. The solution was incubated at 40°C for 175 h. The extent of oxidation was measured by peroxide value using the colorimetric method as reported previously [15].

#### 2.6.4. Bleaching of *β*-Carotene in Linoleic Acid System

Antioxidant activity of *M. longifolia *essential oil and extracts was also assessed by bleaching of *β*-carotene/linoleic acid emulsion system as reported previously [1]. Briefly, a stock solution of *β*-carotene-linoleic acid mixture was prepared by dissolving 0.1 mg *β*-carotene, 20 mg linoleic acid, and 100 mg Tween 40 in 1.0 mL of chloroform (HPLC grade). The chloroform was removed under vacuum in rotary evaporator at 50°C. Then, 50 mL of distilled water saturated with oxygen (30 min, 100 mL min^−1^) was added with vigorous shaking. A 5.0 mL of this reaction mixture was dispensed to test tubes with 200 *μ*L of the essential oil or trans-anethole solution, prepared at 4.0 g L^−1^ concentrations, and the absorbance was immediately (*t* = 0) measured at 490 nm against a blank, consisting of an emulsion without *β*-carotene. Then emulsion was incubated for 50 h at room temperature, and the absorbance was recorded at different time intervals. The same procedure was repeated with BHT and blank. Antioxidant capacities of the fennel essential oils were compared with BHT and blank.

### 2.7. Statistical Analysis

All the experiments were conducted in triplicate unless stated otherwise, and data are presented as mean ± standard deviation (SD). Statistical analysis of the data was performed by Analysis of Variance (ANOVA) using STATISTICA 5.5 (Stat Soft Inc, Tulsa, OK, USA) software, and probability value *P* ≤ 0.05 was considered to denote a statistically significant difference. 

## 3. Results and Discussion

### 3.1. Percentage Yield of Essential Oil and Different Extracts

Yield (g/100 g of dry plant material) of *Mentha longifolia* essential oil and *n*-hexane, dichloromethane, and methanol extracts is given in Table 1. Maximum yield was obtained with methanol (12.60 g/100 g). The minimum yield was obtained with dichloromethane (3.50 g/100 g). The essential oil yield from the aerial parts of *M. longifolia* was found to be 1.07 g/100 g. Nonpolar extract yield (*n*-hexane) was found to be 7.30 g/100 g. Tukey's range test revealed the significant (*P* < 0.05) difference among the extract yield with solvents of different polarities. Differences in yield of extracts from different solvents might be attributed to the availability of extractable component of different polarities.

### 3.2. Essential Oil Composition

**Figure 1:**
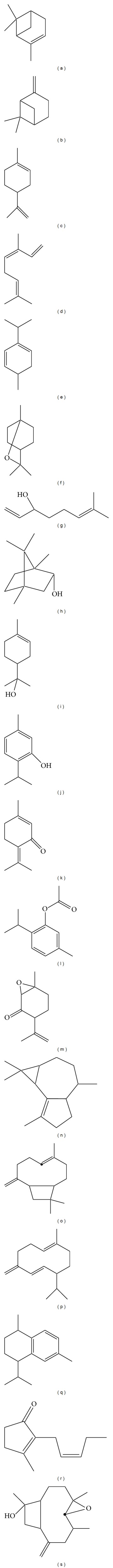
Structure of major compounds detected from *M. longifolia* essential oil. (a) *α*-Pinene; (b) *β*-pinene; (c) limonene; (d) *cis*-*β*-ocimene; (e) *δ*-terpinene; (f) 1,8-cineole; (g) linalool; (h) borneol; (i) *α*-terpineol; (j) thymol; (k) piperitenone; (l) thymol acetate; (m) piperitenone oxide; (n) *α*-gurjunene; (o) *β*-caryophyllene; (p) germacrene D; (q) calamenene; (r) *cis-*jasmone; (s) caryophyllene oxide.

The retention indices, percentage composition, and identification methods for the essential oil of *M. longifolia* are given in Table 2. Nineteen compounds, 96.79% of the total oil, were identified from the oil (Figure 1). The most abundant constituents (>5%) in the essential oil of *M. longifolia *were found to be piperitenone oxide (28.3%), piperitenone (24.9%), germacrene D (8.16%), borneol (5.96%), and *β*-caryophyllene (5.94%). Analyzed essential oil mainly consisted of oxygenated monoterpenes (67.24%) followed by sesquiterpene hydrocarbons (17.19%), monoterpene hydrocarbons (7.31%), and oxygenated sesquiterpenes (5.05%).

The variation in the essential oil composition of* M. longifolia *is reported in the literature from different part of the world [8, 17, 18]. Our results reported the essential oil composition of *M. longifolia* essential oil from South Punjab, Pakistan, where the weather conditions are very hot and dry. Variations in the chemical compositions of essential oil across countries might be attributed to the varied agroclimatic (climatical, seasonal, and geographical) conditions of the regions, isolation regimes, and adaptive metabolism of plants.

### 3.3. Antioxidant Activity

#### 3.3.1. Total Phenolics (TP) and Total Flavonoids (TF) Contents

**Figure 2:**
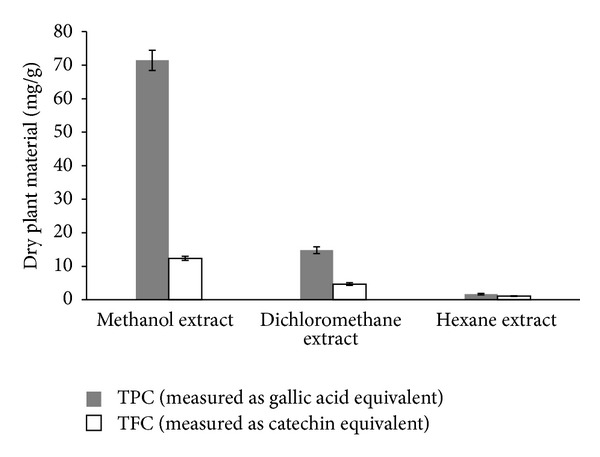
Total phenolics (TP) and total flavonoids (TF) contents of *n*-hexane, dichloromethane, and methanol extracts of *M. longifolia*.

Amount of total phenolics and total flavonoids is given in Figure 2. The highest TP was found in methanol extract (71.43 mg/g acid of dry plant material, measured as gallic equivalent) and the lowest in hexane extract (1.7 mg/g acid of dry plant material, measured as gallic equivalent). Similarly, the amount of TF in methanol, dichloromethane, and hexane extracts was found to be 12.35, 4.7, and 1.1 mg/g acid of dry plant material, measured as catechin equivalent. The effect of different solvent systems on the amount of TP and TF was significant (*P* < 0.05). Methanol has been proven as effective solvent to extract phenolic compounds [6]. 

#### 3.3.2. DPPH Radical Scavenging Assay

The ability of *M. longifolia* essential oil and different extract to donate proton to DPPH free radical and change its color from violet to yellow is accessed in this assay. Concentration of extracts and essential oil scavenging 50% of DPPH radical is shown in Table 3. IC_50_ value ranged from 6.70 to 33.3 *μ*g/mL. Greater IC_50_ value (maximum radical scavenging activity) was observed with methanol extract of *M. longifolia,* and lesser IC_50_ value was recorded with *n*-hexane extract. IC_50_ values of *M. longifolia *essential oil and dichloromethane extracts were found to be comparable with IC_50_ value of the piperitenone, a major compound of *M. longifolia* essential oil. IC_50_ value of methanol extract is significantly (*P* < 0.05) better than hexane and dichloromethane extracts and essential oil and comparable with synthetic antioxidant, BHT. 

#### 3.3.3. Antioxidant Activity Determination in Terms of Inhibition of Linoleic Acid Peroxidation

The antioxidants activity has also been assessed as ability to prevent the oxidation of linoleic acid. Therefore, inhibition of linoleic acid oxidation was also used to assess the antioxidant activity of *M. longifolia* extracts and essential oil. All extracts and essential oil exhibited appreciable inhibition of linoleic acid peroxidation (Table 3) ranging from 9.9 to 91.6%. Methanol extract showed maximum antioxidant activity (91.6%) followed by dichloromethane extract (89.3%) which is comparable with the activity of BHT standard (90.6%). *M. longifolia *essential oil and hexane extract showed weaker antioxidant activity. Polar extract exhibited significantly (*P* ≤ 0.05) higher antioxidant activity than nonpolar extracts which might be due to the higher concentration of TP and TF contents [15]. 

#### 3.3.4. Antioxidant Activity Determination in Terms of Bleaching of *β*-Carotene in Linoleic Acid System

**Figure 3:**
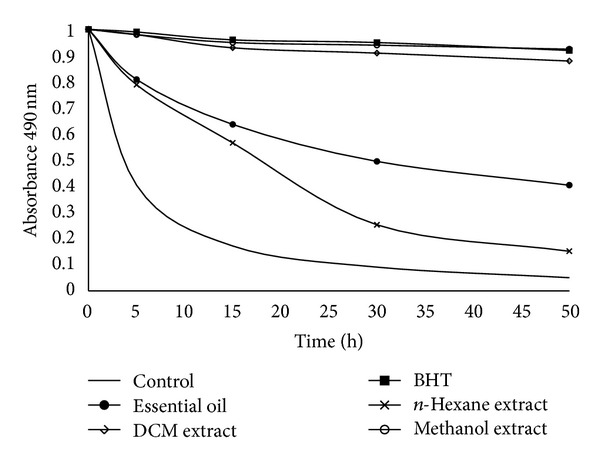
Antioxidant activity of *M. longifolia* essential oil and *n*-hexane, dichloromethane, and methanol extracts in terms of bleaching of *β*-carotene-linoleic acid emulsion.

Bleaching *β*-carotene with linoleic acid system as antioxidant activity of the *M. longifolia* essential oil and extracts is presented in Figure 3. The greater is the effectiveness of an antioxidant, the slower will be the colour depletion. In Figure 3 smaller decline in absorbance of *β*-carotene indicates a lower rate of oxidation of linoleic acid and higher antioxidant activity in the presence of *M. longifolia* methanol and dichloromethane extracts and BHT. Hexane extract and essential oil showed poor antioxidant activity.

## 4. Conclusion

Methanol extracts of *Mentha longifolia* exhibited excellent antioxidant activity and free radical scavenging capacity followed by dichloromethane extract, essential oil, and hexane extract. High TP and TF contents and antioxidant potential of *M. longifolia* extracts lead to its possible use as a food preservative. Moreover, they may be used in pharmaceutical and natural therapies for treatment of oxidative stress. 

## Figures and Tables

**Table 1:** Yield of *M. longifolia* essential oil and hexane, dichloromethane, and methanol extracts.

Samples	Yield (g/100 g)*
Essential oil	1.07 ± 0.10^a^
*n-*Hexane extract	7.30 ± 0.32^c^
Dichloromethane extract	3.50 ± 0.21^b^
Methanol extract	12.60 ± 0.70^d^

*Values are mean ± SD of three samples of *M. longifolia* analyzed individually in triplicate.

Different letters in superscript represent significant (*P* < 0.05) difference within solvents.

**Table 2:** Chemical composition of *M. longifolia* essential oil.

Components^b^	RI^c^	Molecular mass	% Composition^a^	Mode of identification^d^	Quality (%)^e^
Monoterpene hydrocarbons			**(7.31)**		
*α*-Pinene	939	136	0.76 ± 0.06	RT, RI, MS	97
*β*-Pinene	979	136	2.14 ± 0.19	RT, RI, MS	96
Limonene	1029	136	1.80 ± 0.19	RT, RI, MS	94
*cis*-*β*-Ocimene	1037	136	1.98 ± 0.11	RT, RI, MS	97
*δ*-Terpinene	1089	136	0.63 ± 0.04	RI, MS	96
Oxygenated monoterpenes			**(67.24)**		
1,8-Cineol	1031	154	2.00 ± 0.17	RT, RI, MS	98
Linalool	1097	154	0.98 ± 0.10	RT, RI, MS	98
Borneol	1169	154	5.96 ± 0.44	RT, RI, MS	96
*α*-Terpineol	1189	154	1.17 ± 0.09	RT, RI, MS	98
Thymol	1290	150	2.85 ± 0.20	RT, RI, MS	99
Piperitenone	1343	150	24.9 ± 1.34	RT, RI, MS	97
Thymol acetate	1352	192	1.08 ± 0.08	RI, MS	94
Piperitenone oxide	1370	166	28.3 ± 1.6	RT, RI, MS	96
Sesquiterpene hydrocarbons			**(17.19)**		
*α*-Gurjunene	1410	204	1.11 ± 0.18	RI, MS	96
*β*-Caryophyllene	1421	204	5.94 ± 0.32	RT, RI, MS	99
Germacrene D	1485	204	8.16 ± 1.01	RT, RI, MS	99
Calamenene	1540	202	1.98 ± 0.17^d^	RT, RI, MS	98
Oxygenated sesquiterpenes			**(5.05)**		
*cis*-Jasmone	1393	164	1.13 ± 0.11^a^	RT, RI, MS	96
Caryophyllene oxide	1583	220	3.92 ± 0^b^	RT, RI, MS	97

Total			**96.79**		

^a^Values are mean ± standard deviation of three samples of *M. longifolia* essential oil, analyzed individually in triplicate.

^b^Compounds are listed in order of elution from a HP-5MS column; ^c^retention indices relative to C_9_–C_24_ *n*-alkanes on the HP-5MS column; ^d^mode of identifications; RT: identification based on retention time; RI: identification based on retention index; MS: identification based on comparison of MS data compared with those from the NIST02.L and WILEY7n.L mass spectral libraries; ^e^matching percentage with the NIST02.L and WILEY7n.L mass spectral libraries.

**Table 3:** Antioxidant activity of *M. longifolia* essential oil and *n-*hexane, dichloromethane, and methanol extracts.

Samples	Antioxidant activity*	
	DPPH, IC_50_, (*µ*g mL^−1^)	Inhibition of linoleic acid peroxidation (%)
Essential oil	21.8 ± 1.2^c^	37.3 ± 1.3^c^
*n-*Hexane extract	33.3 ± 1.7^d^	9.9 ± 0.7^a^
DCM extract	21.2 ± 1.7^c^	89.3 ± 2.9^d^
Methanol extract	6.70 ± 0.3^a^	91.6 ± 2.3^d^
Piperitenone	22.7 ± 1.5^c^	31.3 ± 2.1^b^
BHT	9.90 ± 0.2^b^	90.9 ± 2.7^d^

*Values are mean ± standard deviation of three samples of each *Thymus* species, analyzed individually in triplicate. Mean followed by different superscript letters in the same column represents significant difference (*P* < 0.05).

NT: not tested.
